# Effect of moderate prenatal ethanol exposure on the differential expression of two histamine H3 receptor isoforms in different brain regions of adult rat offspring

**DOI:** 10.3389/fnins.2023.1192096

**Published:** 2023-06-28

**Authors:** Suzy Davies, Kiana S. Lujan, Ella J. Rappaport, Carlos F. Valenzuela, Daniel D. Savage

**Affiliations:** Department of Neurosciences, University of New Mexico School of Medicine, Albuquerque, NM, United States

**Keywords:** prenatal alcohol (ethanol), histamine H3 receptor, *in situ* hybridization, western blotting, dentate gyrus, cerebral cortex

## Abstract

We have reported that prenatal alcohol exposure (PAE) elevates histamine H_3_ receptor (H3R) agonist-mediated inhibition of glutamatergic neurotransmission in the dentate gyrus. Here, we hypothesized that PAE alters the expression of two prominent H3R isoforms namely, the rH_3A_ and rH_3C_ isoforms, which have differing intrinsic activities for H3R agonists, in a manner that may contribute to heightened H3R function in PAE rats. In contrast to our predictions, we found different effects of sex and PAE in various brain regions with significant interactions between sex and PAE in dentate gyrus and entorhinal cortex for both isoforms. Subsequently, to confirm the PAE-and sex-induced differences on H3R isoform mRNA expression, we developed a polyclonal antibody selective for the rH_3A_ inform. Western blots of rH_3A_ mRNA-transfected HEK-293 cells identified a  ~ 48 kDa band of binding consistent with the molecular weight of rH_3A_, thus confirming antibody sensitivity for rH_3A_ protein. In parallel, we also established a pan-H3R knockout mice line to confirm antibody specificity in rodent brain membranes. Both qRT-PCR and H3R agonist-stimulated [^35^S]-GTPγS binding confirmed the absence of mH_3A_ mRNA and H3 receptor-effector coupling in H3R knockout (KO) mice. Subsequent western blotting studies in both rat and mouse brain membranes were unable to detect rH_3A_ antibody binding at ~48 kDa. Rather, the H3RA antibody bound to a  ~ 55 kDa band in both rat and mouse membranes, including H3R KO mice, suggesting H3RA binding was not specific for H3Rs in rodent membranes. Subsequent LC/MS analysis of the ~55 kDa band in frontal cortical membranes identified the highly abundant beta subunit of ATPase in both WT and KO mice. Finally, LC/MS analysis of the ~48 kDa band from rH_3A_ mRNA-transfected HEK-293 cell membranes was able to detect rH_3A_ protein, but its presence was below the limits of quantitative reliability. We conclude that PAE alters rH_3A_ and rH_3C_ mRNA expression in some of the same brain regions where we have previously reported PAE-induced alterations in H3R-effector coupling. However, interpreting the functional consequences of altered H3R isoform expression was limited given the technical challenges of measuring the relatively low abundance of rH_3A_ protein in native membrane preparations.

## 1. Introduction

Our laboratory has reported spatial and associative learning deficits in adult rat offspring whose mothers consumed moderate quantities of ethanol during pregnancy. These prenatal alcohol exposure (PAE)-induced learning deficits were more apparent with increasingly challenging versions of the behavioral tasks (Sutherland et al., 2000; Weeber et al., 2001; Savage et al., 2002, 2010). Further, baseline input/output physiological responses appeared intact in our PAE rat offspring, whereas more complex activity-dependent changes in synaptic plasticity were more sensitive to PAE (Sutherland et al., 1997; Savage et al., 1998; Varaschin et al., 2010). A series of combined neurochemical and physiologic studies (Perrone-Bizzozero et al., 1998; Savage et al., 2002; Galindo et al., 2004) provided evidence that PAE impairs presynaptic mechanisms associated with activity-dependent potentiation of glutamate release from perforant path terminals in dentate gyrus as one mechanism contributing to the synaptic plasticity and learning deficits observed in PAE rats.

We have since pursued the acquisition of agents with mechanisms of action that target the regulation of neurotransmitter release as potential therapeutic interventions for the synaptic plasticity and learning deficits we had observed in our PAE rats. One such class of so-called “cognition-enhancing” agents are the histamine H3 receptor (H3R) inverse agonists. H3Rs reside primarily on nerve terminals where they inhibit the release of a variety of transmitters including histamine (Arrang et al., 1983), other monoamines (Schlicker et al., 1988, 1989, 1993), acetylcholine (Clapham and Kilpatrick, 1992) and glutamate (Garduño-Torres et al., 2007). Collectively, H3R inverse agonists have been shown to interfere with H3R function, leading to enhanced neurotransmitter release, as well as to exhibit procognitive effects in a variety of animal models of learning and memory [see reviews by Esbenshade et al. (2008), Haas et al. (2008), Brioni et al. (2011), and Nikolic et al. (2014)].

Similar to these prior studies, we reported that the H3R inverse agonist ABT-239 ameliorated both contextual fear conditioning deficits and spatial memory deficits in PAE rats (Savage et al., 2010). Concurrently, in separate groups of control and PAE rats, we reported that ABT-239 reversed PAE-induced deficits in perforant path-dentate gyrus long-term potentiation (LTP) (Varaschin et al., 2010). Further, treatment of saccharin control rats with the selective H3R agonist methimepip mimicked the LTP deficit observed in saline-treated PAE rats (Varaschin et al., 2014). Currently, there is no evidence indicating that H3R function is altered in patients with FASD or that drugs acting at H3Rs affect patients with FASD differently than other patients. However, given our preclinical observations with ABT-239, the ongoing objective of our research program has been to establish a preclinical rationale for considering clinical trials in patients with FASD based on how PAE alters H3R function in our model of moderate prenatal alcohol exposure. Subsequently, our lab investigated whether PAE alters histamine H3R number or function in various regions. Radio-histochemical studies employing the selective H3R antagonist [^3^H]-A349821 indicated that moderate PAE did not affect H3R density in any brain region measured (Varaschin et al., 2018). However, using the selective H3R agonist methimepip to stimulate [^35^S]-GTPγS (GTP) binding as a measure of H3R receptor-effector coupling, we observed a significant enhancement of GTP binding in hippocampal and cerebrocortical regions of PAE rats, particularly at higher agonist concentrations (Varaschin et al., 2018). The functional significance of this heightened H3 receptor-effector coupling response in PAE rats was examined in dentate gyrus slices. The ability of methimepip to decrease the probability of glutamate release from dentate gyrus slices, noted as an increase in the paired-pulse ratio measure, was significantly elevated in PAE rats (Varaschin et al., 2018). Further, the concentrations of methimepip that lowered the probability of glutamate release were similar to the methimepip concentrations that enhanced GTP binding in dentate gyrus of PAE rats.

A closer examination of methimepip’s stimulation of GTP binding indicated that binding increased over a nearly four-order-of-magnitude elevation in agonist concentration in both control and PAE rats (Varaschin et al., 2018). In contrast to agonist concentration-response curves occurring over roughly two orders of magnitude of increasing agonist concentration, which reflect the presence of a single non-interacting receptor binding site, agonist-response curves greater than two orders of magnitude suggest the presence of at least two non-interacting subpopulations of binding sites with differing affinities for binding agonist. Based on prior studies of H3R isoforms measured in expression systems (Drutel et al., 2001; Bakker, 2004; Bongers et al., 2007), we suspected that two functional rH3R isoforms namely, the rH_3A_ and the rH_3C_ isoforms, may be responsible for the methimepip-stimulated GTP binding concentration-response study described above. These two functional H3R isoforms, which are prominent in various higher brain regions (Drutel et al., 2001; Morisset et al., 2001), are splice variants of the same gene. However, due to alternative gene splicing, they differ in their size and pharmacologic properties. The rH_3A_, the full-length (445 amino acid) isoform in rat, displays relatively lower agonist affinity, but higher intrinsic agonist activity, whereas the rH_3C_ isoform (397 amino acids) contains a 48 amino acid deletion in the middle of the third intracellular loop and exhibits higher agonist affinity, but lower intrinsic activity relative to the rH_3A_ (Drutel et al., 2001).

Given that H3R receptor-effector coupling was elevated in PAE rats in the absence of changes in the total H3R number (Varaschin et al., 2018), we hypothesized that PAE causes a net increase in the expression of the lower affinity, but higher intrinsic activity rH_3A_ isoform relative to the higher affinity, but lower intrinsic activity rH_3C_ isoform in PAE rat brain. We first tested this hypothesis by employing *in situ* hybridization to measure the binding density of radiolabeled cDNA probes for each of these two H3R isoforms in various brain regions. However, given the limitations of relating mRNA quantity with protein function (Allen et al., 2013; Moritz et al., 2019), we subsequently developed and characterized a polyclonal antibody to selectively target the rH_3A_ protein and attempted to measure rH_3A_ protein in various brain regions with the prediction that rH_3A_ protein would be elevated in the same brain regions of PAE rats where elevations in H3R agonist-stimulated GTP binding had been observed previously (Varaschin et al., 2018).

## 2. Materials and methods

### 2.1. Housing for Long-Evan rats

The University of New Mexico Health Sciences Center (UNM HSC) Institutional Animal Care and Use Committee (IACUC) approved all of the procedures involving the use of live rodents. All experiments were in compliance with the ARRIVE guidelines. The Long-Evans rats used in these studies were purchased from Envigo Corporation (Indianapolis, IN, USA). Upon arrival at our UNM HSC Animal Resource Facility, the rats were single housed in static micro isolator cages with Envigo Tek-Fresh bedding at 22°C on a reverse 12-h dark/12-h light schedule (lights on from 2100 to 0900 h). They were provided irradiated Teklad Global 2920 Soy Protein Free rodent diet (Envigo) and water (chlorine dioxide treated in water bottles, autoclaved prior to use) *ad libitum*. Female rats arrived at around 6–7 weeks of age (125–150 g) and were approximately 9–10 weeks old at the time of breeding. The males were established breeders, 12 weeks old upon arrival, and 15 to 16 weeks old at the start of the breeding protocol. After one-week acclimation to the animal facility, baseline body weights for each female were obtained prior to the start of the pre-pregnancy drinking phase of the PAE paradigm.

### 2.2. Moderate prenatal ethanol exposure paradigm

The breeding and prenatal alcohol exposure (PAE) procedures for rats employed in this study were the same as described previously (Hamilton et al., 2014; Davies et al., 2023). Pre-pregnancy drinking levels in female rats were evaluated by gradually acclimating them to drinking 5% ethanol in 0.066% saccharin in tap water 4 h each day. The saccharin water contained 0% ethanol on the first and second day, 2.5% ethanol (v/v) on the third and fourth day, and 5% ethanol on the fifth day and thereafter. The drinking tubes were placed in the cages 1 h. after the onset of the dark phase. Daily ethanol consumption was measured using 55 mL volume conical beaded glass test tubes (25 mm × 150 mm; Fisher Scientific International, Waltham, MA, USA) with millimeter graduated markings, topped with a metal sipper tube with a stainless steel ball (Ancare Corporation, Bellmore, NY, USA) inserted in a white #4 rubber stopper (The Plasticoid Company, Elkton, MD, USA). Drinking tubes were filled daily to the 20 mm mark. At the end of each drinking session, the volume consumed was noted as a net change in the mm scale and converted to the volume consumed. The amount of ethanol consumed, expressed as g/kg body weight, was calculated using a daily weight extrapolated from weight data collected at the start and the end of each week. Upon completion of the pre-pregnancy drinking phase, the mean and standard deviation in 5% ethanol consumption from pre-pregnancy Day 5 to Day 14 was calculated. Any females whose drinking was more than one standard deviation below the group mean (usually less than 10% of the group) was removed from the study at this point.

Subsequently, females were assigned to either a saccharin control or 5% ethanol drinking group and matched such that the mean pre-pregnancy ethanol consumption by each group was similar. Females were then placed with alcohol-naïve proven male breeders until pregnant, as indicated by the presence of a vaginal plug. Female rats did not consume ethanol during the breeding procedure, which averaged about 2 days in length. Beginning on Gestational Day 1 (GD1), rat dams were provided saccharin water containing either 0% or 5% ethanol for 4 h each day, from 1000 to 1400 h. The volume of saccharin water provided to the control group (16 mL) was matched to the mean volume of 5% ethanol in saccharin water consumed by the ethanol prenatal treatment group during gestation. Daily four-hour ethanol consumption was recorded for each dam through GD21, after which ethanol consumption was discontinued. Rats were weighed weekly to assess maternal weight gain. At birth, the number of live births were recorded and the pups weighed. Subsequently the litters were culled to 10 pups by Postnatal Day 2 (PD2). Offspring were weaned at PD 23–26, at which time the numbers of each sex was confirmed.

### 2.3. *In situ* hybridization (ISH) studies

**Figure 1:**
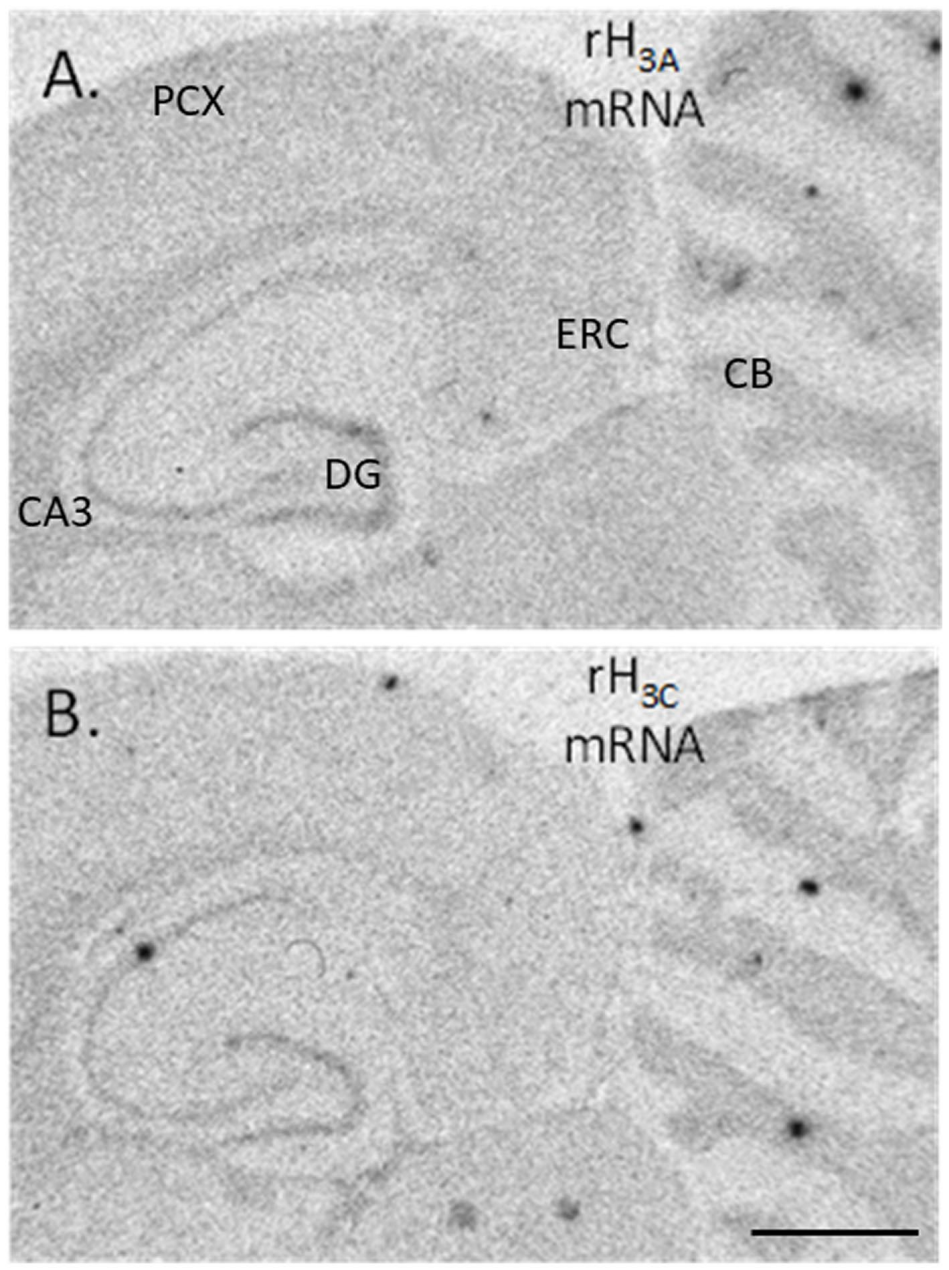
Autoradiograms of hybridized cDNA probes to rH_3A_ mRNA **(A)** and to rH_3C_ mRNA **(B)** present in horizontal sections collected from an unexposed control rat brain. Areas where densitometric measurements were collected are DG – dentate gyrus stratum granulosum, CA3 – hippocampal CA3 stratum pyramidale, CB – cerebellar stratum granulosum, ERC – medial entorhinal cortical layers 2/3, PCX – parietal cortex layers 2/3 (An image of the medial frontal cortex is not shown here, but the pattern of hybridization was identical to the distribution of H3R agonist-simulated GTP binding shown in Figure 5A). The horizontal black line in the bottom right corner of panel **(B)** denotes a distance of 2,500 μm.

Seven pairs of seven-month-old control and PAE rats of each sex were sacrificed by decapitation and their brains dissected, frozen in isopentane chilled in a dry ice/methanol bath, and then stored in airtight containers at −80°C until sectioning. Sixteen-μm-thick horizontal sections were collected at Figure 107 (Bregma: – 4.60 mm; Interaural: 5.40 mm) according to the Paxinos and Watson rat stereotaxic atlas (Paxinos and Watson, 1998) and stored in airtight containers at-80°C until incubation. Oligonucleotide probes (Thermo Fisher Scientific, Waltham, MA, USA) were designed to specifically recognize rH_3A_ and rH_3C_ mRNA, as previously described (Drutel et al., 2001). The probes (2.5–5 pmol) were labeled with 62.5 μCi of [^35^S]-deoxyadenosine 5′-α (−thio) triphosphate (Perkin Elmer Inc. Boston, MA, USA), at their 3′ ends using terminal deoxynucleotidyl transferase for 2 h. Non-incorporated nucleotides were removed by purification through QIAquick columns (Qiagen Sciences, Germantown, MD, USA). Hybridization was carried out using quintuplicate sections from each rat brain at 52°C for 20 h in a humidified chamber. After post-hybridization washing, the sections were dried and then placed along with ^14^C standards in x-ray cassettes to expose Hyperfilm (GE HealthCare Technologies Inc. Chicago, IL, USA) for 14 days at 4°C. The film was then developed manually in Kodak D-19 (1:1, 18°C) for 2 min, fixed and then mounted onto microscope glass slides. Microdensitometric measurements of rH_3A_ and rH_3C_ mRNA were performed using Media Cybernetics Image Pro Plus® on an Olympus BH-2 microscope. An optical density standard curve, expressed in picoCuries/10^5^μm^2^, was established based on autoradiograms of ^14^carbon standards. Measurements were made in six different brain regions (see Figure 1) at 3.125X total magnification. The density of rH_3A_ mRNA expression and rH_3C_ mRNA expression were each corrected against an off-section background density in each brain region measured in each rat.

### 2.4. Rat H3A polyclonal antibody design and characterization in HEK-293 cells

**Figure 2:**
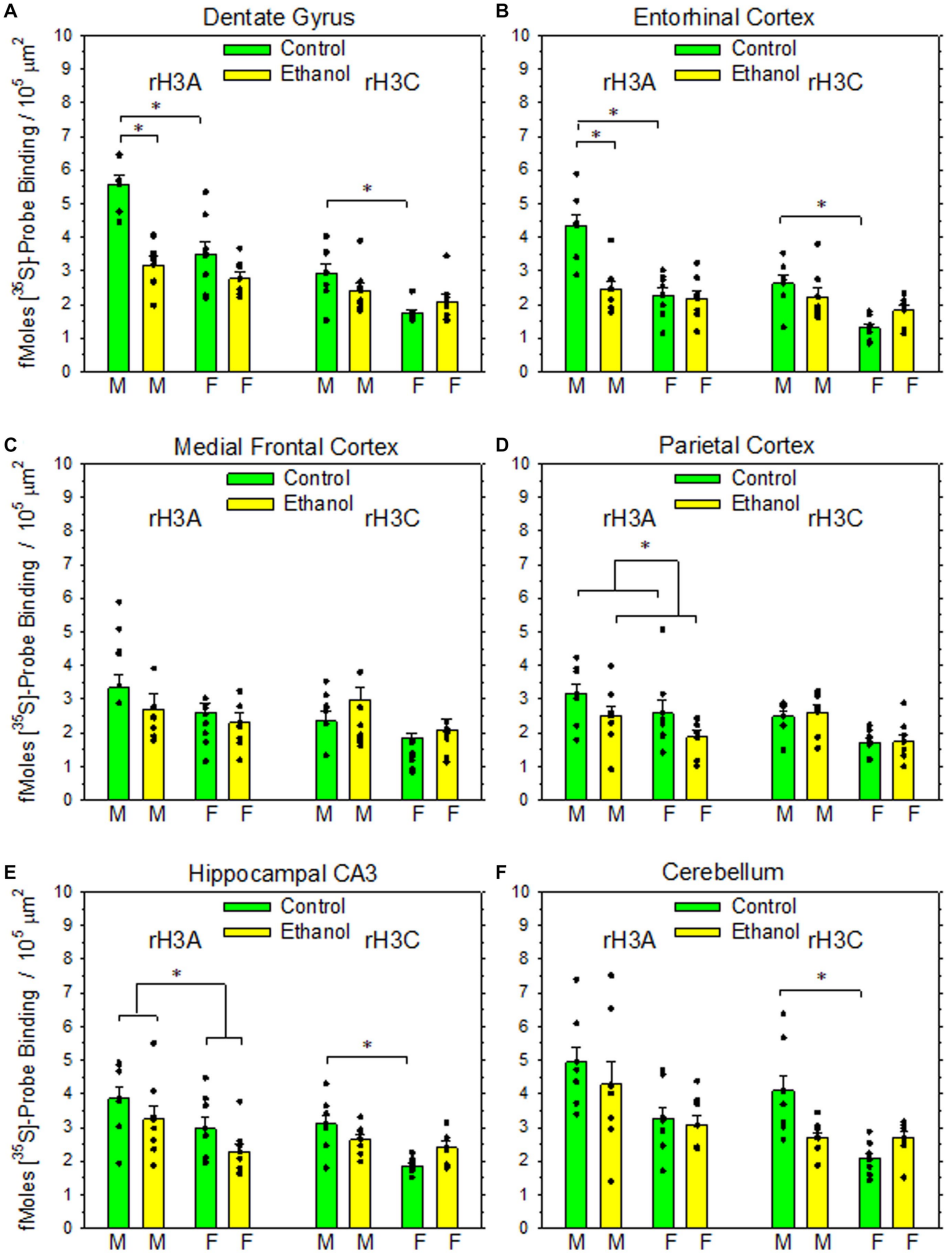
Impact of PAE on rH_3A_ mRNA and rH_3C_ mRNA expression in six rat brain regions. Data bars represent the mean ± SEM density of [^35^S]-labelled cDNA probe binding, expressed as fMoles bound/10^5^ μm^2^ from seven pairs of control and PAE rats of each sex. **(A)** A two-way ANOVA of rH3A probe binding in dentate gyrus revealed a significant interaction between prenatal treatment group and sex (*F*_1,27_ = 9.02, *p* = 0.006). A Holm-Sidak All Pairwise Multiple Comparisons Procedure indicated a significant effect of sex within the control group (*t* = 5.30, *p* < 0.001), but not within the PAE group (*t* = 1.05, *p* = 0.303). A significant effect of prenatal treatment group was also observed between males (*t* = 6.08, *p* < 0.001) but not between females (t = 1.84, *p* = 0.077). A Kruskal-Wallis One-Way ANOVA on Ranks on the rH3C probe binding data indicated a significant difference based on sex with the control group (*q* = 4.52, *p* < 0.05). **(B)** A two-way ANOVA of rH3A probe binding in entorhinal cortex revealed a significant interaction between prenatal treatment group and sex (*F*_1,27_ = 12.2, *p* = 0.002). The Holm-Sidak procedure indicated a significant effect of sex within the control group (*t* = 5.67, *p* < 0.001), but not within the PAE group (*t* = 0.737, *p* = 0.467). A significant effect of prenatal treatment group was also observed between males (*t* = 5.23, *p* < 0.001), but not between females (*t* = 0.297, *p* = 0.768). A two-way ANOVA of rH3C probe binding revealed a significant interaction between prenatal treatment group and sex (*F*_1,27_ = 5.45, *p* = 0.027). The Holm-Sidak procedure indicated a significant effect of sex within the control group (*t* = 4.73, *p* < 0.001), but not within the PAE group (*t* = 1.431, *p* = 0.163). The comparisons between prenatal treatment within sex were not significant for either sex. **(C)** A two-way ANOVA of rH3A probe binding in medial frontal cortex revealed no significant effect of prenatal treatment (*F*_1,27_ = 1.65, *p* = 0.209), sex (*F*_1,27_ = 2.34, *p* = 0.138) or a significant interaction (*F*_1,27_ = 0.222, *p* = 0.641). Likewise, a two-way ANOVA of rH3C binding revealed no significant effect of prenatal treatment (*F*_1,27_ = 3.96, *p* = 0.057), sex (*F*_1,27_ = 0.003, *p* = 0.955) or significant interaction (*F*_1,27_ = 0.075, *p* = 0.786). **(D)** A two-way ANOVA of rH3A probe binding in parietal cortex revealed a significant main effect of prenatal treatment (*F*_1,27_ = 5.09, *p* = 0.032) but not sex (*F*_1,27_ = 3.78, *p* = 0.062). A K-W ANOVA on Ranks on the rH3C probe binding data indicated no significant effects based on prenatal treatment or sex. **(E)** A two-way ANOVA of rH3A probe binding in hippocampal CA3 revealed a significant main effect of sex (*F*_1,27_ = 7.53, *p* = 0.010), but not prenatal treatment (*F*_1,27_ = 3.73, *p* = 0.064). A two-way ANOVA of rH3C probe binding data revealed a significant interaction between prenatal treatment and sex (*F*_1,27_ = 7.32, *p* < 0.001). A H-S Comparisons procedure indicated a significant effect of sex within the control group (*t* = 4.708, *p* < 0.001), but not within the PAE group (*t* = 0.880, *p* = 0.386). No effects based on prenatal treatment were observed in either males (*t* = 1.787, *p* < 0.085) or females (*t* = 2.04, *p* = 0.051). **(F)** A K-W ANOVA on Ranks on the rH3A probe binding data in cerebellum indicated no significant differences based on prenatal treatment or sex. A K-W ANOVA on Ranks on the rH3C probe binding data indicated a significant effect of sex within the control group (*q* = 5.65, *p* < 0.05).

We contracted GenScript (Piscataway, NJ, USA) to develop a polyclonal antibody against a fourteen amino acid sequence within the third cytoplasmic loop of the rH_3A_ isoform, a sequence not present in other functional rH3 isoforms (see Figure 2). A FASTA search indicated this sequence is only present in rH_3A_ of different species and not present in other receptors. Antibody specificity was characterized using human HEK-293 cells cultured in T175 cell culture flasks (Avantor™, Radnor, PA, USA) in DMEM media containing 0.584 g/L glutamine (Sigma-Aldrich, Inc., St. Louis, MO, USA) 5% penicillin–streptomycin (Thermo Fisher Scientific) and 10% Fetal Bovine Serum (FBS, Sigma-Aldrich, Inc.) in a humidified atmosphere with 5% CO_2_ until 65–75% confluence on the day of transfection. As described by Bakker et al. (2006), the cells were transfected with cDNAs encoding each of six rH3 isoforms (A-F) from GenScript (cDNA sequences are provided at the GenScript website: www.genscript.com/search?q=rat+histamine+receptor+isoforms&g=orf), labeled with a C-terminal DYKDDDK tag (FLAG tag) detectable *via* immunoblotting, using the LipofectaminePlus method, according to the manufacturer’s protocol (Thermo Fisher Scientific). Briefly, on the day of transfection, the cells were trypinized with 0.05% trypsin–EDTA (Gibco™, Thermo Fisher Scientific) and washed with media to collect the HEK-293 cells and centrifuged for 5 min at 300× *g* to pellet the cells. The cDNAs (rH_3A_-_C_: 46 μg and rH3_D-F_: 90 μg) and Lipofectamine (92–180 μL) were diluted in Opti-MEM media without serum (Sigma-Aldrich, Inc.) and, after 5 min incubation, were mixed together and incubated for 20 min at room temperature to allow complex formation to occur. The complex was added directly to each well containing cells and mixed gently by rocking the plate back and forth. The media was changed after 6 h. The cells were incubated for 24–48 h and harvested by centrifugation.

Protein was extracted from the transfected HEK 293 cells with isotonic homogenization buffer (320 mM sucrose, 10 mM Tris HCl, 1 mM NaVO_4_, 5 mM NaF, 1 mM EDTA, 1 mM EGTA, 1 mM DTT) containing 1× protease and phosphatase inhibitors (Sigma-Aldrich, Inc.). The cells were then homogenized in a glass Dounce homogenizer with six up and down strokes each using a loose fitting and then a tight fitting pestle. The resulting homogenate was then centrifuged at 1000× *g* for 6 min at 4°C. The supernatant (S1) was then centrifuged again at 10,000× *g* for 15 min at 4°C. The resulting P2 fraction was resuspended in homogenization buffer. Proteins were quantified using a BCA Protein Assay Kit (Pierce, Rockford, IL, USA) according to manufacturer’s instructions. The fraction was aliquoted, flash frozen in liquid nitrogen and stored at −80°C until use.

Subsequently, 5 μg of sample protein were loaded onto a 4–12% NuPage gradient gel (Invitrogen™, Thermo Fisher Scientific Inc.) and run at 150 V for 2 h. The proteins were transferred onto a PVDF membrane (Millipore Sigma, Burlington, MA, USA). The membranes were blocked using I-block (Applied Biosystems, Foster City, CA, USA) in 1X PBS at room temperature for 1 h, then incubated with the GenScript anti-rH_3A_ (H3RA) polyclonal antibody (1:5000) and the GenScript anti-FLAG tag (1:2500, A00187) overnight at 4°C in I-block plus 0.1% Tween-20. Membranes were then washed three times for 10 min in 1X PBS-T, incubated in secondary antibodies at 1:15,000–1:20,000 dilution (LI-COR Biosciences, Lincoln, NE, USA) in I-block containing PBS plus 0.1% Tween-20 and 0.01% SDS for 1 h at room temperature. Membranes were then scanned using the LI-COR Odyssey Infrared Imaging System using the 700 nm wavelength channel to visualize the Tag and Actin and the 800 nm channel for H3RA antibody binding. The optical densities were quantitated using LI-COR Odyssey Application Software version 3.0.

### 2.5. Breeding of H3R knockout mice

As another strategy for confirming H3AR specificity, we acquired the B6.129P2-*Hrh3*^tm1Tw^*l*J line of transgenic mice from The Jackson Laboratory (JAX stock #023433; Bar Harbor, ME, USA). These mice carry a targeted mutation of the *Hrh3*, histamine receptor H3R gene, with a cassette containing a neomycin resistance gene replacing a 0.7-kilobase region covering part of the first intron and the 5′ end of the second exon of the gene (Toyota et al., 2002). Jackson Labs provided male and female heterozygote breeding pairs for our experiments. Breeding pairs were maintained in the UNM HSC Breeding Barrier. The mice were kept in ventilated standard cages with autoclaved recycled cardboard bedding with low lights on from 0600 to 1800 h and provided irradiated Teklad Global 2920 Soy Protein Free rodent diet (Envigo) and sterilized water pouches *ad libitum*. Offspring mouse genotype was determined with tail biopsies using real-time PCR with specific probes designed for HrH3 (Transnetyx, Cordova, TN, USA). The three genotypes generated were Wild type (WT – mH3R^+/+^), heterozygous (HT – mH3R^+/−^) and pan H3R Knockout (KO – mH3R^−/−^).

### 2.6. RNA extraction and qRT-PCR from mouse frontal cortex

Medial frontal cortex was collected from five pairs of ~five-month-old adult WT (all female) and KO (two male and three female) mice, flash frozen and stored at −80°C until RNA extraction. Total RNA was extracted using TRIzol™ (Invitrogen™, Thermo Fisher Scientific). RNA quality was determined using a NanoDrop 2000 (Thermo Fisher Scientific), using absorbance at 260, 280 and 230 nm. One μg of total RNA was reverse-transcribed using SuperScript III reverse transcriptase (Invitrogen™, Thermo Fisher Scientific) following the manufacturer’s protocol and qRT-PCR carried out with a Model 7500 fast real-time PCR system (Applied Biosystems™, Thermo Fisher Scientific) using PowerUp SYBR Green master mix (Thermo Fisher Scientific). Primer sequences for qRT-PCR for mouse mRNAs were made by Invitrogen (Thermo Fisher Scientific) to detect mouse H3R Transcript Variant 1 (Forward primer sequence: CTTCCTCGTGGGTGCCTTC, Reverse primer sequence: CAGCTCGAGTGACTGACAGG) and the GAPDH gene (Forward primer sequence: TGTGATGGGTGTGAACCACGAGAA, Reverse primer sequence: GAGCCCTTCCACAATGCCAAAGT). For each sample, the GAPDH cycling time (CT) was subtracted from mH3R CT and then the relative expression of mH3R was determined using the comparative 2-ΔCt method (Livak and Schmittgen, 2001). All 2-ΔCt values were normalized to the mean of WT mH3R expression and the normalized values expressed as the mean ± SEM.

### 2.7. Immethridine-stimulated [^35^S]-GTPγS (GTP) binding studies In mouse brain

As described above for the ISH studies in Section 2.3, four ~ten-week-old mice, two male and two females from each of the three genotypes, were sacrificed, their whole brains dissected, frozen in isopentane and stored in airtight containers at −80°C until sectioning. Eight-μm-thick cryostat sections were collected in the sagittal plane corresponding to Figure 113 (Lateral: - 1.44 mm) in the Paxinos & Franklin stereotaxic atlas of mouse brain (Paxinos and Franklin, 2013), thaw-mounted onto pre-cleaned Superfrost-Plus® microscope slides and stored at −80°C in airtight containers until incubation. Tissue sections were pre-incubated for 10 min in incubation buffer (50 mM Tris–HCl, 100 mM NaCl, 5 mM MgCl_2_, 0.2 mM EGTA, 2 mM GDP, 1 mM DTT, and 100 nM DPCPX; pH 7.4 at 25°C), and then incubated with 100 pM [^35^S]-GTPγS (Perkin Elmer, specific activity = 1,250 Ci/mmole) for 90 min at 25°C with the H3R agonist immethridine (3 μM) in the absence or presence of 30 μM unlabeled iodopotentidine (IPP), a H3R antagonist. After incubation, sections were rinsed twice for 15 s each in ice-cold incubation buffer, dipped in ice-cold distilled water, air-dried, and vacuum desiccated overnight. Sections were then exposed to Kodak Biomax MR film for 4 days, and the film developed in Kodak D-19 (1:1, 18°C), fixed and then mounted onto microscope glass slides. Measurements of [^35^S]-GTPγS binding were performed using Media Cybernetics Image Pro Plus® on an Olympus BH-2 microscope at 3.125X total magnification. An optical density standard curve, expressed in picoCuries/10^5^ μm^2,^ was established based on autoradiograms of ^14^carbon standards. Immethridine-stimulated [^35^S]-GTPγS binding was expressed as percent of GTP binding above GTP binding in the presence of iodopotentidine. The mean ± the SEM percent agonist-stimulated GTP binding was computed for each brain region for each genotype.

### 2.8. Western blotting of P2 membranes from rat and mouse frontal cortex

Protein was extracted from frontal cortical brain tissue of ~seven-week-old female untreated control rats or from ~eleven-week-old WT and H3R KO mice of both sexes using isotonic homogenization buffer containing protease and phosphatase inhibitors as described for HEK-293 cells in Section 2.4. We ran varying amounts of sample protein (2–50 μg) on 4–12% gradient gels and optimized the primary antibody concentrations as follows: GenScript H3RA (1:5000), and β-Actin (1:80,000, Sigma-Aldrich Inc.) as the reference protein. Secondary antibodies used were donkey anti-rabbit IRDye® 800CW (1:20,000, LI-COR Biosciences) for H3RA, and goat anti-mouse IRDye® 630RD (1:40,000, LI-COR Biosciences) for β-actin. The fluorescence images of the western blots were analyzed as described previously (Section 2.4).

### 2.9. Western blotting and LC/MS of the ~55 kDa band of P2 membranes from frontal cortex

#### 2.9.1. Western blotting

Frontal cortical tissue was dissected from a seven-week-old-female rat, and eleven-week-old WT and KO mice of both sexes, and protein extracted using isotonic homogenization buffer containing protease and phosphatase inhibitors as described for HEK-293 cells in Section 2.4. The resulting P2 pellet was kept at −80°C until shipment to Kendrick Laboratories (Madison, WI, USA). Upon arrival, the pellets were dissolved in 250 μL of SDS Boiling Buffer without reducing agents. All samples were heated in a dry bath for 10 min at 95°C before the sample protein concentrations were determined using the BCA assay (Smith et al., 1985). Samples were then diluted to 3.0 μg/μl in SDS Boiling Buffer with reducing agents and heated in a digital dry bath at 95°C for 10 min. Samples were then further diluted to 1.5 mg/mL in SDS Boiling Buffer with reducing agents before loading. One dimensional SDS slab gel electrophoresis was carried out under reducing conditions according to the method of Laemmli (1970) as modified by O'Farrell (1975). Twenty-five μg of sample were loaded into wells in 10% acrylamide slab gels (0.75 mm thick). SDS slab gel electrophoresis was carried out for about 4 h at 15 mA/gel. The following proteins (Millipore Sigma) were used as molecular weight standards: Myosin (220,000), phosphorylase A (94,000), catalase (60,000), actin (43,000), carbonic anhydrase (29,000), and lysozyme (14,000). These standards are visible in lanes on images of the Coomassie blue-stained PVDF membranes (images not shown).

After slab gel electrophoresis, the gels for blotting were placed in transfer buffer (10 mM Caps, pH 11.0, 10% methanol) and transblotted onto PVDF membranes overnight at 200 mA. The blot was stained with Coomassie Brilliant Blue R-250. The blot was then blocked for 2 h in 5% non-fat dry milk (NFDM) diluted in Tween-20 tris buffer saline (TTBS). The blot was then incubated overnight in primary antibody (GenScript H3RA, 1:25,000) in 2% NFDM TTBS and rinsed 3 × 10 min in TTBS. The blot was then placed in secondary antibody (anti-Rabbit IgG-HRP [SeraCare, Milford, MA, USA, Cat# 5220–0337, Lot# 10440068] diluted 1:20,000 in 2% NFDM TTBS) for 2 h, rinsed as above, treated with ECL, and exposed to x-ray film for 3 min. The ~55 kDa bands of interest were excised from the corresponding gel and sent to Dr. Costel Darie at the Clarkson University Protein Core Facility (Potsdam, NY, USA) for protein identification by LC MS/MS.

#### 2.9.2. Protein digestion and peptide extraction

Proteins that were separated by SDS-PAGE/2D-PAGE and stained by Coomassie dye were excised, washed and the proteins from the gel were treated according to published protocols (Channaveerappa et al., 2017; Aslebagh et al., 2018; Mihăşan et al., 2018; Wormwood et al., 2018; Lux et al., 2019). Briefly, the gel pieces were washed in high purity, high performance liquid chromatography HPLC grade water, dehydrated and cut into small pieces and destained by incubating in 50 mM ammonium bicarbonate, 50 mM ammonium bicarbonate/50% acetonitrile, and 100% acetonitrile under moderate shaking, followed by drying in a speed-vac concentrator. The gel bands were then rehydrated with 50 mM ammonium bicarbonate. The procedure was repeated twice. The gel bands were then rehydrated in 50 mM ammonium bicarbonate containing 10 mM DTT and incubated at 56°C for 45 min. The DTT solution was then replaced with 50 mM ammonium bicarbonate containing 100 mM iodoacetamide for 45 min in the dark, with occasional vortexing. The gel pieces were then re-incubated in 50 mM ammonium bicarbonate/50% acetonitrile, and 100% acetonitrile under moderate shaking, followed by drying in speed-vac concentrator. The dry gel pieces were then rehydrated using 50 mM ammonium bicarbonate containing 10 ng/μL trypsin and incubated overnight at 37°C under low shaking. The resulting peptides were extracted twice with 5% formic acid/50 mM ammonium bicarbonate/50% acetonitrile and once with 100% acetonitrile under moderate shaking. Peptide mixture was then dried in a speed-vac, solubilized in 20 μL of 0.1% formic acid / 2% acetonitrile.

#### 2.9.3. NanoLC–MS/MS

The peptides mixture was analyzed by reverse phase nanoliquid chromatography (LC) and MS (LC–MS/MS) using a NanoAcuity UPLC (Micromass/Waters, Milford, MA) coupled to a Q-TOF Xevo G2 mass spectrometer (Micromass/Waters, Milford, MA), according to published procedures (Channaveerappa et al., 2017; Aslebagh et al., 2018; Mihăşan et al., 2018; Wormwood et al., 2018; Lux et al., 2019). Briefly, the peptides were loaded onto a 100 μm x 10 mm NanoAquity BEH130 C18 1.7 μm UPLC column (Waters, Milford, MA) and eluted over a 60 min gradient of 2–80% organic solvent (ACN containing 0.1% FA) at a flow rate of 400 nL/min. The aqueous solvent was 0.1% FA in HPLC water. The column was coupled to a Picotip Emitter Silicatip nano-electrospray needle (New Objective, Woburn, MA). MS data acquisition involved survey MS scans and automatic data dependent analysis (DDA) of the top six ions with the highest intensity ions with the charge of 2+, 3+ or 4+ ions. The MS/MS was triggered when the MS signal intensity exceeded 250 counts/s. In survey MS scans, the three most intense peaks were selected for collision-induced dissociation (CID) and fragmented until the total MS/MS ion counts reached 10,000 or for up to 6 s each. The entire procedure used was previously described (Channaveerappa et al., 2017; Aslebagh et al., 2018; Mihăşan et al., 2018; Wormwood et al., 2018; Lux et al., 2019). Calibration was performed for both precursor and product ions using 1 pmol GluFib (Glu1-Fibrinopeptide B) standard peptide with the sequence EGVNDNEEGFFSAR and the monoisotopic doubly-charged peak with m/z of 785.84.

#### 2.9.4. Data processing and protein identification

The raw data were processed using ProteinLynx Global Server (PLGS, version 2.4) software as previously described (Channaveerappa et al., 2017; Aslebagh et al., 2018; Mihăşan et al., 2018; Wormwood et al., 2018; Lux et al., 2019). The following parameters were used: background subtraction of polynomial order 5 adaptive with a threshold of 30%, two smoothings with a window of three channels in Savitzky–Golay mode and centroid calculation of top 80% of peaks based on a minimum peak width of 4 channels at half height. The resulting pkl files were submitted for database search and protein identification to the in-house Mascot server (Matrix Science, London, UK)^1^ for database search using the following parameters: databases from NCBI (*mouse and histamine H3A receptor*), parent mass error of 0.5 Da with 1 ^13^C, product ion error of 0.8 Da, enzyme used: trypsin, three missed cleavages, propionamide as cysteine fixed modification and Methionine oxidized as variable modification. To identify the false negative results, we used additional parameters such as different databases or organisms, a narrower error window for the parent mass error (1.2 and then 0.2 Da) and for the product ion error (0.6 Da), and up to two missed cleavage sites for trypsin. In addition, the pkl files were also searched against in-house PLGS database version 2.4^2^ using searching parameters similar to the ones used for Mascot search. The Mascot and PLGS database search provided a list of proteins for each gel band. To eliminate false positive results, for the proteins identified by either one peptide or a mascot score lower than 25, we verified the MS/MS spectra that led to identification of a protein.

### 2.10. Western blotting and LC/MS of the ~48 kDa band of P2 membranes from HEK-293 cells

HEK-293 cells were transfected with rH_3A_ mRNA and Lipofectamine only, as described in Section 2.4. Protein was extracted and varying concentrations of protein (20–50 μg) were run on a 4–12% gel which was stained with Coomassie Brilliant blue R-250. Western blotting was conducted as described above in Section 2.4 and the ~48 kDa band was excised from the corresponding gel and sent for LC–MS analysis as described in Section 2.9 above.

### 2.11. Statistical procedures

**Figure 3:**
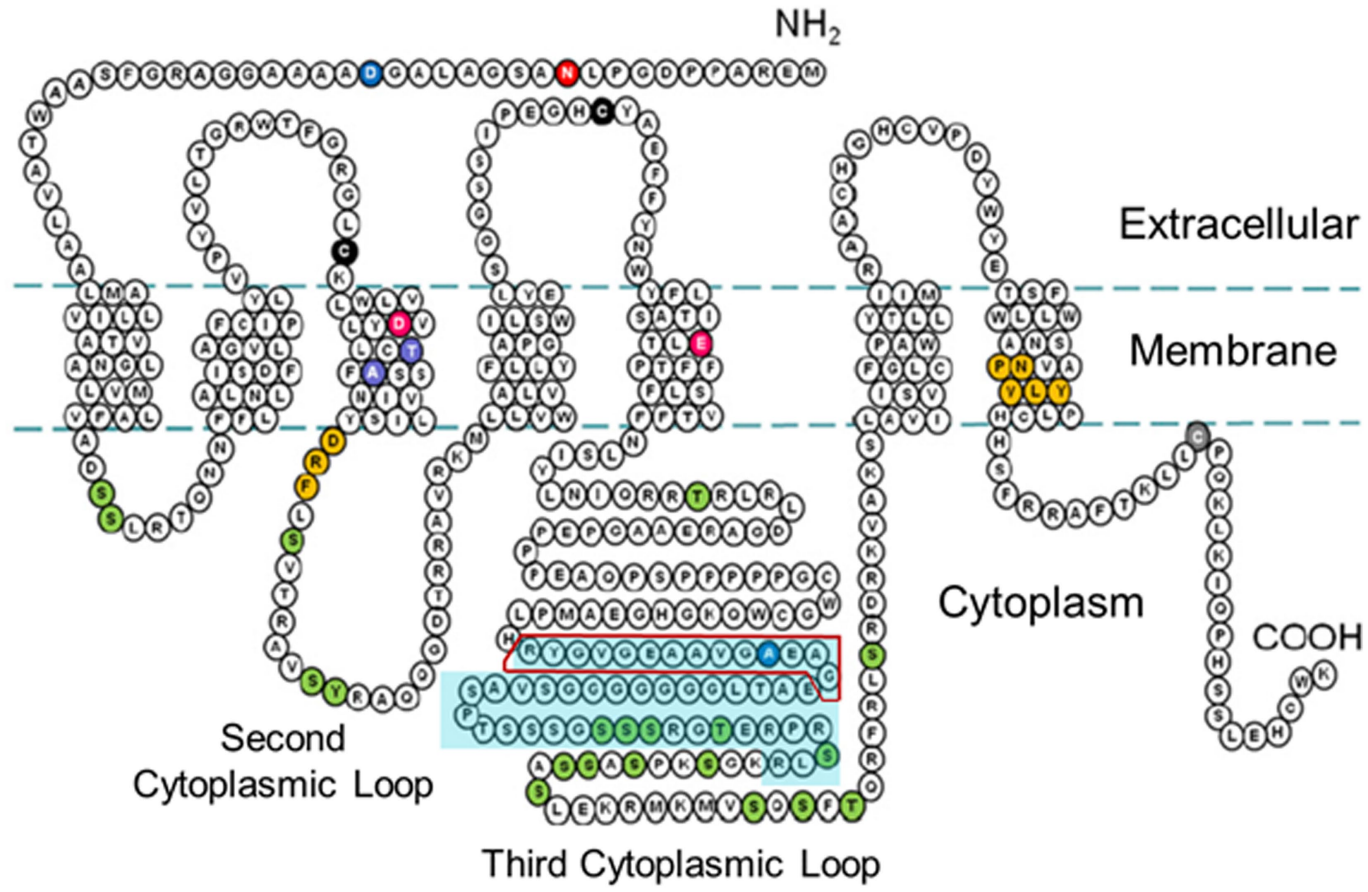
Two-dimensional schematic diagram of the amino acid sequence of the rH3A isoform [adapted from the amino acid sequence diagram in Nieto-Alamilla et al., 2016] illustrating the seven transmembrane spanning regions and the three cytoplasmic loops. The turquoise-shaded area within the third cytoplasmic loop denotes the 48 amino acid region not present in the rH_3B_ and rH_3C_ isoforms. The red box outline within that region denotes the 14 amino acid sequence used to raise the GenScript polyclonal antibody to the rH3A isoform.

**Figure 4:**
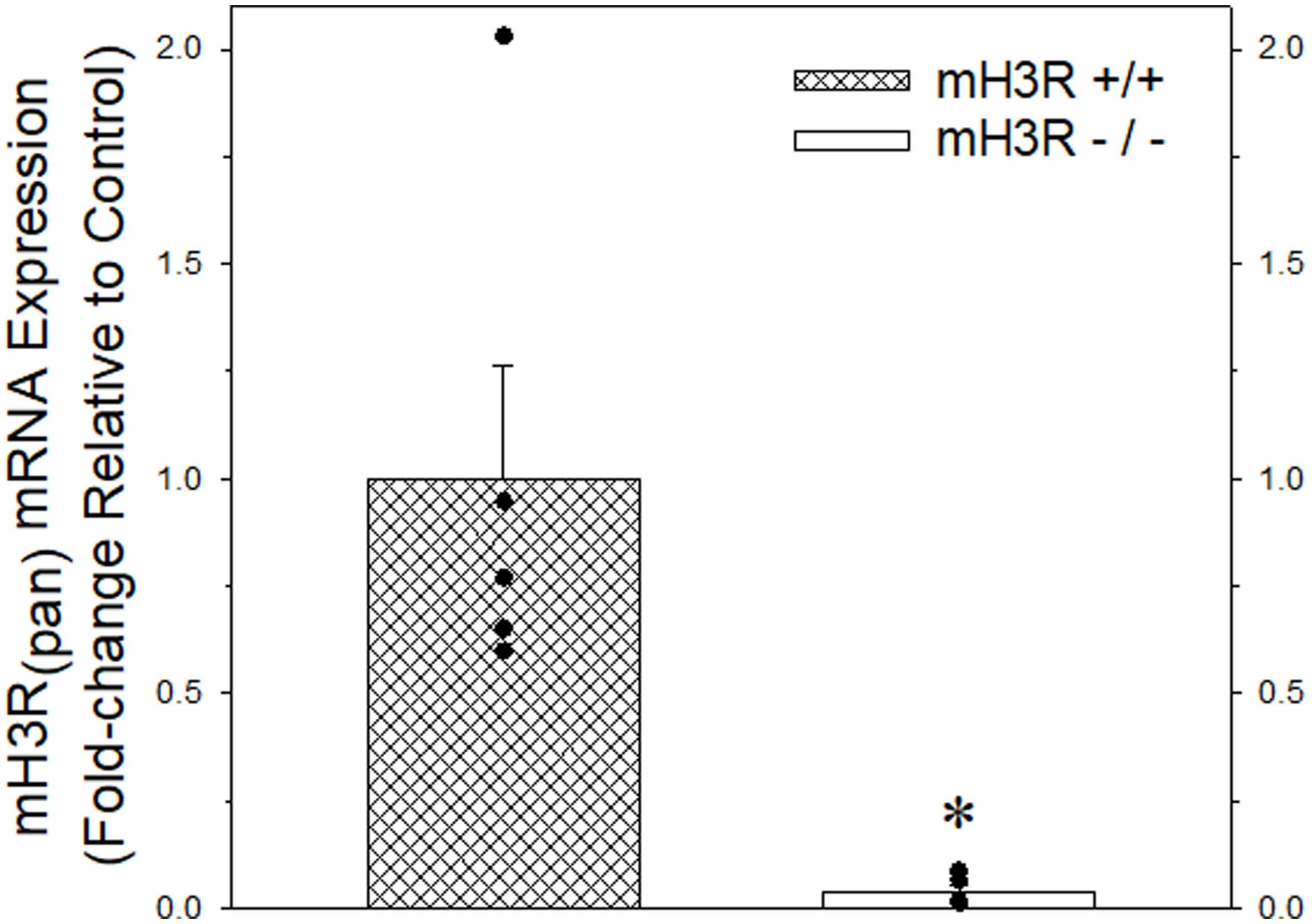
Quantitative real-time PCR analysis of pan-mH3R mRNA expression in P2 membranes prepared from frontal cortex of five pairs of WT (course cross hatching bar) and mH3R KO (white bar) mice. Data bars represent the mean ± SEM of mRNA expression normalized to the mean of the WT group. A Mann–Whitney Rank Sum Test indicated a significant reduction in pan-H3R mRNA expression, denoted by the asterisk, in the mH3R KO group compared to the WT control group (*T* = 40.0, *p* = 0.008).

**Figure 5:**
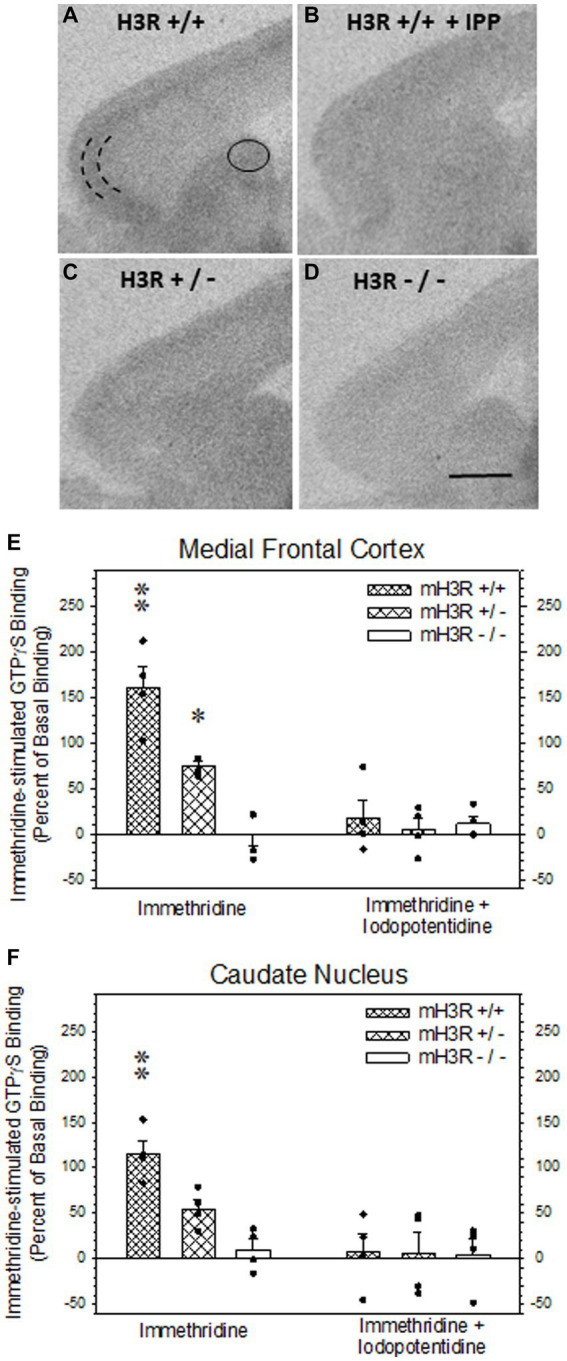
H3R agonist-stimulated [^35^S]-GTPγS (GTP) binding in sagittal sections of frontal mouse brain. **(A,B)** H3R agonist (3 μM immethridine)-stimulated GTP binding in WT mH3R^+/+^ mouse in the absence **(A)** and in the presence of 30 μM of the H3R antagonist iodopotentidine (IPP, **B**). **(C,D)** H3R agonist-stimulated GTP binding in the absence of IPP in HT mH3R^+/−^ **(C)** and the KO mH3R^−/−^ mouse genotypes. The curved parallel lines in panel **(A)** denote where microdensitometric measurements were performed in the external pyramidal layer of the medial frontal cortex **(D)** and within the oval circle in dorsal medial caudate nucleus **(A)**. **(E,F)** Effects of genotype and incubation condition on immethridine stimulated GTP binding in mouse brain. The data bars in each graph represent the mean ± SEM H3R agonist-simulated GTP binding, expressed as percent of binding above the GTP binding in the presence of added IPP, from four mice in each of the three genotypes. A two-way ANOVA of the GTP binding data in medial frontal cortex **(E)** revealed a significant interaction between genotype and the presence of H3R antagonist IPP in the incubation medium (*F*_2,23_ = 13.6, *p* < 0.001). A Holm-Sidak All Pairwise Multiple Comparisons Procedure indicated a significant effect of incubation condition in the WT group (*t* = 6.80, *p* < 0.001) and the HT group (*t* = 3.33, *p* = 0.004) but not in the KO group (*t* = 0.582, *p* = 0.568). Within genotype in the absence of IPP, there was a significant difference between WT and HT (*t* = 4.08, *p* < 0.001) and between WT and KO (*t* = 7.66, *p* < 0.001), denoted by the double asterisk, and between HT and KO (*t* = 3.59, *p* = 0.002), denoted by the single asterisk. No differences between genotypes were noted in the presence of IPP. A two-way ANOVA of the GTP binding data in dorsal medial caudate nucleus **(F)** also indicated a significant interaction between genotype and the presence of H3R antagonist IPP in the incubation medium (*F*_2,23_ = 4.64, *p* = 0,024). The Holm-Sidak procedure indicated a significant effect of incubation condition in the WT group (*t* = 4.52, *p* < 0.001), but not the HT group (*t* = 2.06, *p* = 0.054) or the KO group (*t* = 0.221, *p* = 0.828). Within genotype in the absence of IPP, there was a significant difference between WT and HT (*t* = 2.53, *p* = 0.021) and between WT and KO (*t* = 4,42, <0.001), denoted by the double asterisk, but not between HT and KO (*t* = 1.89, *p* = 0.075). Again, there were no differences between genotype in the presence of IPP.

All statistical procedures and graphical illustrations were performed using SigmaPlot® 11 (Systat Software Inc., San Jose, CA, USA). All data are expressed as the mean ± SEM with a *p* value <0.05 deemed as statistically significant. A Mann–Whitney Rank Student’s two-tailed t-tests was used to compare differences in maternal weight gain and pup birthweight and Mann–Whitney rank sum test was used to test differences in litter size (Table 1). Two-way ANOVAs were used to test for differences in the individual rH3A mRNA and rH3C mRNA expression data in each brain region unless the data was not normally distributed, in which case a Kruskal-Wallis ANOVA on ranks was employed (Figure 3). A Mann–Whitney rank sum test was used to compare the qRT-PCR data (Figure 4). A two-way ANOVA was used to test differences in GTP binding data in the absence and presence of the H3R antagonist iodopotentidine (Figure 5).

**Table 1:** Daily ethanol consumption by dams in the 5% ethanol group, resulting serum ethanol concentration and impact of moderate prenatal ethanol exposure on pregnancy outcome measures.

	Saccharin control	5% Ethanol
Number of dams (*n*)	11	10
Daily gestational ethanol consumption (g/kg)	NA	1.81 ± 0.05
Serum ethanol concentration (mg/dL)	NA	25.1 ± 3.3^a^
Maternal weight gain (g)	103 ± 5	88 ± 4*
Number of live births (n)	10.9 ± 0.2	9.6 ± 0.6
Pup birthweight (g)	7.85 ± 0.33	8.61 ± 0.45

a Samples collected 2 h after the introduction of drinking tubes (Davies et al., 2023).

* Denotes maternal weight gain data significantly less that control (*t* = 2.66, *p* = 0.015).

## 3. Results

### 3.1. Paradigm outcome measures

Rat dams in the 5% ethanol group consumed a mean of 1.81 ± 0.05 grams of ethanol each day during gestation (Table 1). In a separate set of rat dams, this level of consumption produced a mean serum ethanol concentration of 25.1 ± 3.3 mg/dL when sampled 2 h after the introduction of drinking tubes (Davies et al., 2023). This moderate prenatal ethanol exposure paradigm resulted in a 14% reduction in maternal weight gain that was statistically significant. However, the reduction in maternal weight gain did not significantly affect offspring litter size or offspring birth weight (Table 1).

### 3.2. *In situ* hybridization studies in rat brain

As illustrated in Figure 1, both rH_3A_ mRNA and rH_3C_ mRNA were heterogeneously distributed across brain regions in patterns similar to the distribution of H3Rs and H3R agonist-stimulated [^35^S]-GTPγS binding (Varaschin et al., 2018). The density of rH_3A_ binding appears to be greater than the rH_3C_ signal. Figure 3 summarizes the impact of PAE and sex on the expression of rH_3A_ mRNA and rH_3C_ mRNA in six brain regions. No prenatal treatment or sex-related effects were consistently observed across the six brain regions analyzed. Significant interactions between prenatal treatment and sex were observed for rH_3A_ mRNA expression in the dentate gyrus (3A) and entorhinal cortex (3B) and for rH_3C_ RNA expression in entorhinal cortex (3B) and hippocampal CA3 region (3E). In each of these four cases, along with non-parametric assessments of rH_3C_ mRNA in dentate gyrus (3A) and cerebellum (3F), a sex-related effect was detected in control but not PAE offspring. Further, a significant main effect of sex was noted for rH_3A_ mRNA in the hippocampal CA3 region (3E). A significant main effect of prenatal treatment was only observed in the parietal cortex (3D) and a significant difference based on prenatal treatment was observed for males but not females in dentate gyrus (3A) and entorhinal cortex (3B). No significant effects were observed in the medial frontal cortex (3C).

### 3.3. Characterization of rH3A antibody specificity in HEK-293 cells

**Figure 6:**
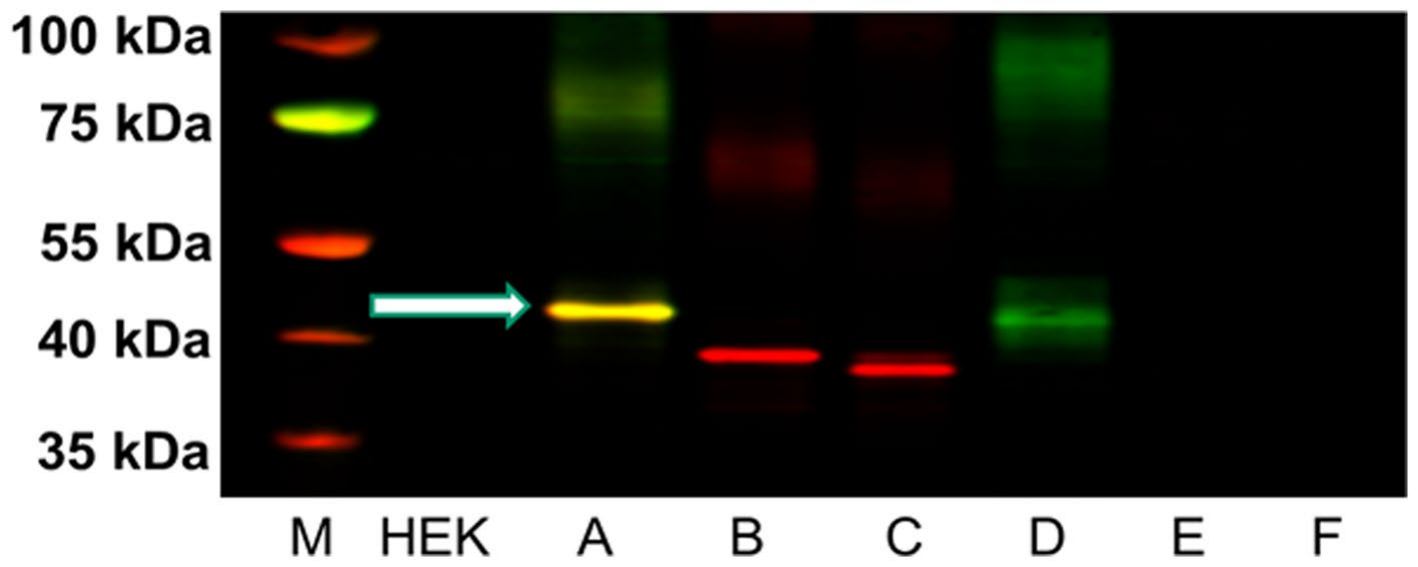
Western blot analysis of five μg of P2 membrane fractions of HEK-293 cells transfected with cDNA for each of the six rH3 isoforms (Lanes A–F). The GenScript antibody recognized protein at ~48 kDa for both rH_3A_ (yellow band in Lane A) and rH_3D_ (green band in Lane D). The red bands in lanes B and C correspond to the anti-FLAG Tag antibody binding to the lower molecular weight rH_3B_ and rH_3C_ isoforms. The non-functional rH3_D-F_ isoforms were present in too low an abundance to be detected by the anti-FLAG tag (Lanes D–F). The yellow band in lane A denotes that both rH3A and the FLAG Tag were detected. M denotes the molecular weight marker bands.

We developed a polyclonal antibody against a fourteen amino acid sequence within the third cytoplasmic loop of the rH_3A_ isoform, a sequence not present in other functional rH3 isoforms (Figure 2) for use in our Western blotting studies. Figure 6 illustrates a representative western blot of HEK-293 P2 samples prepared from six different overexpressing H3R isoform derived cell lines (A–F). Green bands represent H3RA antibody binding, red bands are binding by the Tag peptide, and the yellow band represents binding by both the H3RA antibody and the Tag peptide. The yellow band detected at ~48 kDa in Lane A representing the rH3A-overexpressing HEK-293 cells of Figure 6, confirms the specificity of the GenScript polyclonal antibody for detecting rH_3A_ protein. While sufficient H3R protein was available to visualize Tag peptide binding (red bands) for rH_3B_ (Lane B) and kDa for rH_3C_ (Lane C), the H3RA antibody did not recognize these proteins, as evidenced by the absence of yellow bands. Note that the band positions in Lane B and Lane C are consistent with the shorter amino acid sequences for rH_3B_ (413 aa) and for rH_3C_ (397 aa). However, as the non-functional rH_3D_ (Bakker et al., 2006) has a similar amino acid sequence to rH_3A_, a faint green band of H3RA antibody binding was noted. Anti-FLAG tag antibody labeling was not observed for H3R isoforms D–F (Lanes D–F) due to the relatively low abundance of these isoforms compared to the A, B, and C isoforms.

### 3.4. Quantitative RT-PCR analysis of pan mH3R expression in mouse frontal cortex

As a second approach for confirming antibody specificity, we obtained heterozygote pan H3R transgenic mouse breeding pairs and then generated and confirmed mouse offspring with the WT (mH3R^+/+^), HT (mH3R^+/−^) and KO (mH3R^−/−^) genotypes. Subsequently, we conducted a qRT-PCR analysis of pan-mH3R mRNA expression in medial frontal cortical membranes prepared from WT and KO mice. GAPDH mRNA expression, whose expression was not different between WT and KO mice, was used as the reference gene. However, as shown in Figure 4, we observed a significant 25-fold reduction in pan H3R mRNA expression in KO mice compared to WT mice.

### 3.5. Immethridine-stimulated [^35^S]-GTPγS (GTP) binding in mouse brain

We also employed H3R agonist-stimulated [^35^S]-GTPγS (GTP) binding as a measure of H3R function in all three genotypes. Autoradiograms of immethridine-stimulated GTP binding in sagittal sections of WT mice (Figure 5A) showed a relatively higher level of agonist-stimulated GTP binding in external pyramidal layer of medial frontal cortex with more moderate and diffuse levels of binding in the dorsomedial caudate nucleus, as well as other regions of the cerebral cortex (Figure 5A) and hippocampal formation (not shown). H3R agonist-stimulated GTP binding was virtually abolished in the presence of the H3R antagonist iodopotentidine (IPP; Figure 5B). A fainter degree of agonist-stimulated GTP binding was noted in the medial frontal cortex and caudate nucleus of sections collected from a H3R HT mouse (Figure 5C) and was not present in sections from H3R KO mice (Figure 5D). The H3R agonist-stimulated GTP binding data, expressed as percent increase in GTP binding above the mean binding in the presence of IPP from four mice of each of the three genotypes is summarized for medial frontal cortex (Figure 5E) and caudate nucleus (Figure 5F). A two-way ANOVA of the GTP binding in medial frontal cortex revealed a significant interaction between genotype and the presence of IPP in the incubation medium (Figure 5E). Agonist-stimulated GTP binding was not different among the three genotypes in the presence of IPP nor between the GTP binding in the absence and presence of IPP in the H3R KO group, indicating the absence of H3R agonist-stimulated GTP binding in the H3R KO mice. However, agonist-stimulated GTP binding was significantly greater in the HT group compared to the KO group and significantly greater in the WT group compared to the two other genotypes. Similar results were observed in the caudate nucleus except the agonist-stimulated GTP binding in the HT group was not significantly greater than the H3R KO group (Figure 5F).

### 3.6. Western blotting of P2 membranes from rat frontal cortex

**Figure 7:**
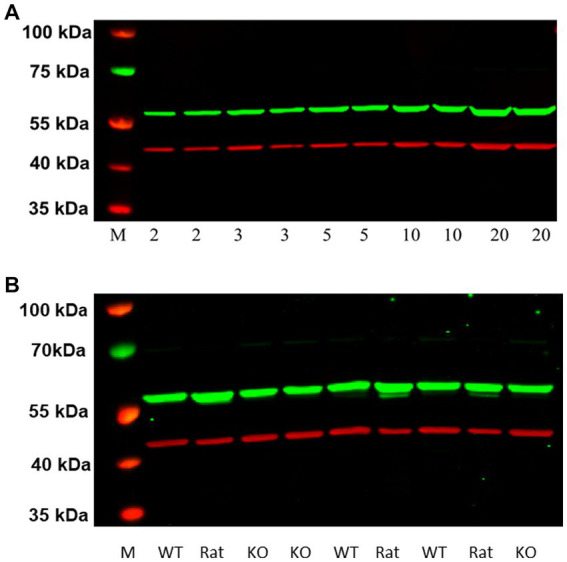
**(A)** Western blot analysis of increasing protein concentrations of P2 membranes from rat frontal cortical tissue **(A)**. Visualization of the GenScript antibody (green band at ~55 kDa) and the reference protein β actin (red band at ~42 kDa) can be seen. M denotes the molecular weight marker bands. We did not detect a ~ 48 kDa band of H3RA antibody binding here, as seen in HEK-293 cells (Figure 6). **(B)** Comparison of western blots of frontal cortical membranes from rat, and WT and H3R KO mice. As observed before for rat membranes, the green band at ~55 kDa was also observed in both WT and KO mice, suggesting that the ~55 kDa band is not H3R protein.

As illustrated in Figure 7A, western blots of increasing protein concentrations of P2 membranes prepared from rat frontal cortical tissue identified a single higher molecular weight band (~55 kDa) in contrast to the ~48 kDa band observed with HEK-293 cells overexpressing rH_3A_ (Figure 6). Subsequent studies employing higher quantities of protein (30, 50 and 100 μg) along with higher concentrations of the primary (1:5,000–1:500) or secondary (1:20,000–1:5,000) antibody failed to detect a ~ 48 kDa band in rat frontal cortex. Our preliminary interpretation of these results was that either the rH_3A_ isoform undergoes post-translational modifications in native membranes that increase its molecular weight or, that the H3RA antibody was binding to another, possibly higher abundance, protein. Subsequently, with confirmation of the mouse H3R phenotypes, we repeated this experiment comparing frontal cortical membranes from rat as well as WT and KO mice. As illustrated in Figure 7B, we observed that the H3RA antibody labeled a ~ 55 kDa band in all three sample types suggesting the ~55 kDa band was not rH_3A_ protein.

### 3.7. LC/MS analysis of the ~55 kDa band of P2 membranes from rodent frontal cortex

**Figure 8:**
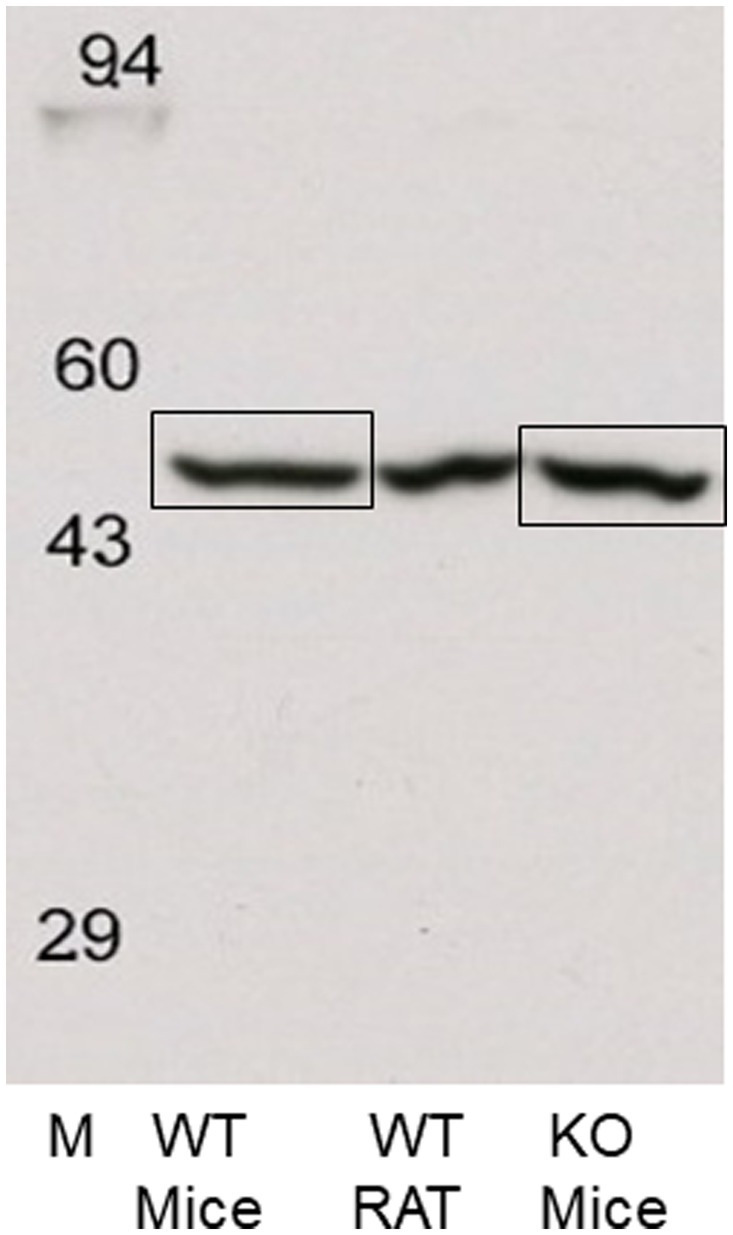
Western blots of rat and WT and H3R KO mouse frontal cortical membranes performed by Kendrick Laboratories using chemiluminescence to visualize the ~55 kDa band. The ~55 kDa bands from the WT and H3R KO mice, denoted by the boxes, were excised from their corresponding gels and sent to the Clarkson Proteomic Facility for LC/MS identification. The ~55 kDa in both WT and KO samples was identified as the beta-subunit of the highly abundant ATPase protein. No mH3A protein was detectable in these samples.

Subsequently, frontal cortical membranes of rat and H3R WT and KO mice were prepared and sent to Kendrick Laboratories along with our GenScript H3RA antibody. Western blotting was performed as before except that Enhanced chemiluminescence (ECL) was used to detect the bands on X-ray film. As before, a single band of antibody binding was observed at ~55 kDa in all three tissue types. The ~55 kDa bands were excised from the mouse WT and KO samples, as illustrated in Figure 8, and sent to the Clarkson Proteomic Facility for protein identification. LC/MS analysis of the ~55 kDa band identified the presence of the highly abundant β-subunit of ATPase in both WT and KO mice, with no evidence of the rH_3A_ protein isoform.

### 3.8. LC/MS analysis of the ~48 kDa band of P2 membranes from HEK-293 cells

**Figure 9:**
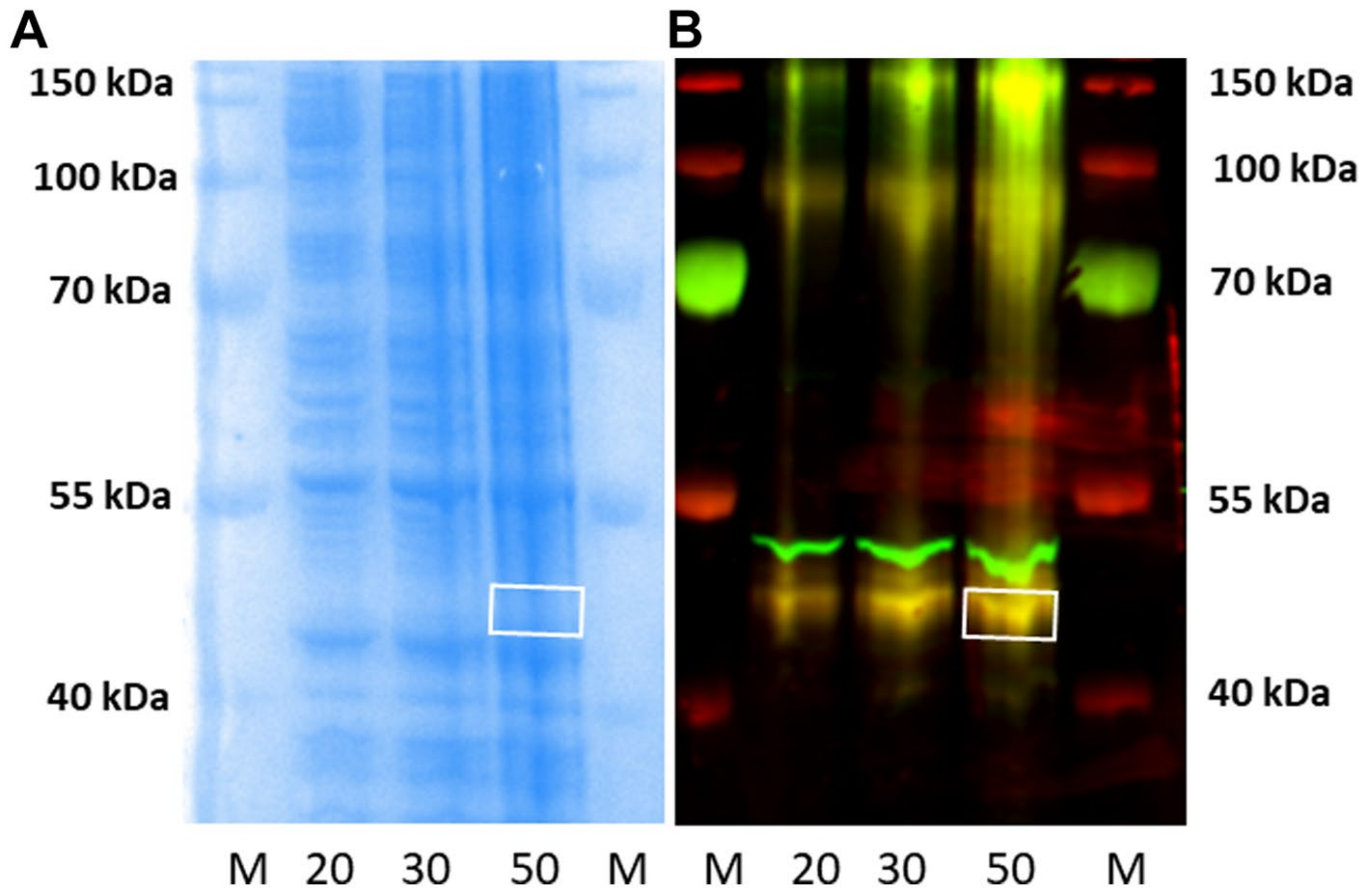
Western blotting of P2 membranes from HEK-293 cells overexpressing rH_3A_ were run in our laboratory, as before. Panel **(A)** is the Coomassie-stained gel corresponding to the western blot shown in panel **(B)**. M denotes the molecular weight marker lanes, and the numbers (20, 30, and 50) denote the μg amount of P2 membranes applied to the gels. Yellow bands, denoting H3RA antibody binding to rH3A protein was observed in all three tissue lanes, not only at ~48 kDa but also at ~100 kDa and ~ 150 kDa suggesting the presence of dimers and trimers of rH3A protein **(B)** Another protein, not observed previously using lower amounts of HEK-293 sample protein, appeared above the rH3A band, but below the ~55 kDa band of non-specific binding observed in rodent membranes (Figures 7, 8). This band was not labeled by anti-FLAG antibody. The ~48 kDa band from the 50 μg lane was excised from the corresponding gel [denoted by the white box in panel **(A)**] and subsequently confirmed by LC/MS to be rH_3A_ protein. However, its detection was below the limit of quantitative reliability (LOQ).

Subsequently, western blotting experiments were repeated in our laboratory, as described in Section 3.3 above, using higher amounts (20–50 μg) of P2 membranes from rH_3A_ mRNA-transfected HEK 293 cells than used in earlier studies (Figure 6). Figure 9A illustrates a Coomassie-stained gel of increasing amounts of protein. Figure 9B is the fluorescence image of the western blot illustrating increasing amounts of rH_3A_ protein at ~48 kDa (yellow bands). In addition, we noted yellow bands near the ~100 kDa and ~ 150 kDa molecular weight markers, suggesting the possibility of rH_3A_ dimers and trimers in this preparation (Figure 9B). Finally, the antibody also bound to another protein (green band) just above the ~48 kDa rH_3A_ band, but below the ~55 kDa band observed in western blots of rodent membranes (Figure 7). This protein, whose identity is uncertain at this point, had not been observed in earlier studies using lower amounts (5 μg) of HEK-293 membrane protein. Subsequently, the ~48 kDa band was excised from the 50 μg lane of the gel (white box in Figure 9A) and sent to the Clarkson Laboratory where LC/MS analysis detected the presence of rH_3A_ protein from the excised band. However, the level of rH_3A_ protein detected was below the limit of quantitative reliability (LOQ), even in rH_3A_ overexpressing HEK-293 cells, suggesting that it would not be possible to quantitate rH_3A_ in rodent membranes.

## 4. Discussion

The salient observation from this study is that moderate PAE is associated with variable alterations in the expression of two H3R mRNA isoforms in dentate gyrus and some cerebral cortical and cerebellar brain regions of adult offspring. While no consistent pattern of mRNA alterations were noted for either H3R mRNA isoform, a prevailing sex-related effect was noted for one or both isoforms in five of the six brain regions studied. In all of these cases, mRNA expression was significantly greater in males than females and, with the exception of the hippocampal CA3 region (Figure 3E), only in control rats. A significant main effect of prenatal treatment was only observed in the parietal cortex (Figure 3D), but significant treatment differences were also observed in the dentate gyrus (Figure 3A) and entorhinal cortex (Figure 3B) in males only.

Examining whether the H3R mRNA alterations were consistent with what would have been predicted based on prior H3R agonist-stimulated GTP binding data in some of these brain regions (Varaschin et al., 2018) is problematic at this point. First, the H3R-agonist-stimulated GTP binding data reported previously was based entirely on studies in female rats. Nevertheless, given that elevations in GTP binding were noted previously in dentate gyrus, parietal cortex, medial frontal cortex and cerebellum, one prediction based on the greater intrinsic activity of rH_3A_ compared to rH_3C_ would have been a PAE-induced elevation in rH_3A_ relative to rH_3C_ in these four brain regions, at least in female rat brain. However, prenatal treatment effects were not observed in any of the six brain regions of female rats. Prenatal treatment effects were observed for males in dentate gyrus and parietal cortex, but not medial frontal cortex. However, the elevation in rH_3A_ mRNA expression occurred in control rats, not PAE rats, as would have been predicted from the H3R agonist-stimulated GTP binding data. Whether the effect of sex on rH_3A_ and rH_3C_ expression could have been predicted based on H3R agonist-stimulated GTP binding in male rats awaits completion of ongoing studies of H3R agonist-stimulated GTP binding in male control and PAE offspring. Thus, whether a PAE-induced shift in H3R mRNA isoform expression relates to diminished H3R agonist responsiveness is unclear. Further, there are many cases where there are either no or even negative correlations between mRNA expression and protein expression data (Moritz et al., 2019) that limit interpreting the functional implications of these alterations in mRNA expression.

Given the limitations of interpreting changes in mRNA expression relative to measures of H3R function, it was critically important to quantitate H3R isoform protein expression in brain regions where PAE altered H3R mRNA expression (Figure 3) as well as “downstream” measures of H3R function (Varaschin et al., 2018). While there are a number of commercially available pan-H3R antibodies, our goal, given our working hypothesis, was to design an antibody that specifically binds to rH_3A_ protein without detecting the other two functional rH3Rs, namely the rH_3B_ and rH_3C_. To this end, we developed a polyclonal antibody based on a fourteen amino acid segment in the third cytoplasmic loop of the rH_3A_ that is not present in either the rH_3B_ or rH_3C_ isoforms (see Figure 2). Subsequently, we were able to demonstrate H3RA specificity for binding rH_3A_ protein in membranes prepared from overexpressing HEK-293 cells (Figure 6). In addition to H3RA antibody development, we generated a transgenic line of pan-mH3R knockout mice to further test H3RA antibody specificity and confirmed the virtual absence of pan-H3R mRNA (Figure 4) and H3 agonist receptor-effector coupling (Figure 5) in H3R KO mouse brain.

Going forward, we predicted that rH_3A_ protein expression would be elevated in PAE rat brain, consistent with the original hypothesis of this study. However, extensive efforts to visualize rH_3A_ protein expression in rat membranes by western blotting were frustrated by the inability to detect the same ~48 kDa band of antibody binding as had been seen in rH_3A_ mRNA-overexpressing HEK-293 cells (Figure 6). Increasing the amount of protein sample or the concentrations of primary and secondary antibody were not successful. We also acquired a pan-H3R antibody from MyBioSource that was used in conjunction with the GenScript H3RA antibody to explore, different immunoprecipitation procedures for enriching H3R protein from tissue preparations using a protocol modified from Cotney and Noonan (2015), and tried to visualize protein both by fluorescence and chemiluminescence detection procedures. We also switched from β-actin (~42 kDa) to the α-_1_ subunit of Na^+^/K^+^ ATPase (110 kDa), as a better reference protein for membrane-associated proteins given that β-actin labeling may have interfered with visualization of H3 receptor isoforms. However, none of these manipulations were successful for detecting the rH_3A_ protein isoform in rat brain membrane preparations. Our tentative conclusion from these studies was that rH_3A_ protein may exist in relatively low abundance in comparison to other receptor proteins and not amenable to quantitation by standard immunological techniques.

While we were unable to visualize H3RA antibody binding to a ~ 48 kDa band in rat membranes, the antibody did label a different band at ~55 kDa (Figure 7A). We subsequently ran western blots of medial frontal cortex from rat, and WT and H3R KO mice which showed the presence of the ~55 kDa band in all three preparations (Figure 7B), indicating that the ~55 kDa the band was not H3R protein. A subsequent LC/MS analysis of the ~55 kDa bands excised from WT and KO mice gels (Figure 8) confirmed no detectable H3R protein in the WT mouse, but identified the highly abundant beta subunit of ATPase protein in both genotypes.

These latter results highlight one of the challenges concerning antibody specificity, particularly when attempting to quantitate relatively low-abundance proteins in native tissues. At the outset of this study, we opted to develop a polyclonal antibody, which are generally less expensive and faster to produce, and they can also recognize multiple epitopes of a single protein antigen, thus providing higher sensitivity for detecting lower abundance proteins. However, polyclonal antibodies can have problems with batch to batch variability. They also have a higher chance of cross-reactivity due to recognition of multiple epitopes that can also cause antibody binding to other protein antigens of lower affinity but present in higher concentrations; such as with the beta subunit of ATPase in the ~55 kDa band from rodent membranes in our studies (Figures 7, 8) and an as yet unidentified protein observed with higher sample protein amounts from HEK-293 cells (Figure 9). The issue of specificity becomes more problematic when increasing the amount of sample protein, primary or secondary antibody in an attempt to detect the protein of interest.

Two alternative approaches for detecting rH_3A_ were considered in lieu of a polyclonal antibody. The first was to consider developing a monoclonal antibody to rH_3A_. In general, monoclonal antibodies are more specific for target protein antigens, given that only a single epitope of an antigen is recognized, thus, they have lower cross-reactivity and also greater homogeneity between batches. However, monoclonal antibodies require significantly more time to produce and, thus, are more expensive to manufacture. Given the relatively lower sensitivity for protein detection with a single binding epitope coupled with the low relative abundance of H3R protein, we were not confident of greater success at visualizing rH_3A_ protein with a monoclonal antibody than we had been with the rH_3A_ polyclonal antibody. A second alternative approach that was considered, given that we have visualized pan-H3R binding with the H3R antagonist [^3^H]-A349821 (Varaschin et al., 2018), and routinely measure H3R function by H3R agonist-stimulated [^35^S]-GTPγS binding (Figure 5), was to radiolabel our GenScript polyclonal antibody. Such an approach would have greatly increased the sensitivity of antibody binding reaction providing the radiolabeling procedure did not affect antibody affinity or specificity. However, significant disadvantages to this approach include the relatively short half-lives of radioisotopes that could be employed in these studies along with the prohibitively high cost of purchasing many radioisotopes in recent years.

In conclusion, we demonstrate here sex-and/or moderate PAE-related alterations in the expression of at least two H3R mRNA isoforms in rat brain regions where we have previously demonstrated elevated H3R function (Varaschin et al., 2018). However, the mRNA alterations observed in Figure 3 were variable across brain regions, were sex-related in some cases and, where present, the prenatal treatment effects were in the opposite direction from what had been predicted from H3R agonist-stimulated GTP binding data reported previously. Further, interpreting the functional consequences of altered H3R mRNA isoform expression in PAE rats is limited given the inability to assess these changes at the receptor isoform protein level. Thus, we were unable to confirm whether a PAE-induced alteration in the differential expression of specific H3R isoforms is one mechanism contributing to the heightened H3 receptor-effector coupling and H3R function in dentate gyrus and cerebral cortex of PAE rats. Our studies here also highlight the challenge of quantitating proteins that exist in very low abundance of native tissues. Indeed, while the rH_3A_ protein isoform was detectable in rH_3A_-overexpressing HEK-293 cells, rH_3A_ protein was present in too low of an abundance in rodent membranes to be reliably quantitated by the methods employed here. Further, most all prior reports measuring H3R isoform expression and examining the function of different H3R isoforms have relied on overexpression systems, such as HEK-293 cells, to generate enough H3R protein to conduct studies using standard biochemical approaches.

While we were unable to confirm whether PAE differentially alters different H3R isoforms in a manner that would alter H3R function, it is important to point out that PAE may alter a variety of other “downstream mechanisms” that could modify H3R receptor affinity and/or H3 receptor-effector coupling in a manner that alters H3R receptor function, independent of whether H3R number or isoform expression have been altered. For example, H3Rs, like other G protein-coupled receptors (Gainetdinov et al., 2004; Gurevich et al., 2012), undergo desensitization and downregulation in the presence of H3R agonists (Garduño-Torres and Arias-Montaño, 2006; Osorio-Espinoza et al., 2014; Montejo-López et al., 2016). While the mechanisms that mediate desensitization have not been investigated with H3Rs, other GPCRs are desensitized via multiple mechanisms including the activation of phospholipases, protein kinases and protein phosphatases, which can modulate receptor affinity and function of GPCRs (Sorensen et al., 2002; Suh et al., 2013). It is interesting to note that prior studies have shown reductions in the activity of phospholipase β1 and phospholipase A2 (Allan et al., 1997), protein kinase C (Perrone-Bizzozero et al., 1998) and extracellular signal-regulated kinase (Samudio-Ruiz et al., 2009) in hippocampal formation of PAE rodents. Whether any of these enzymes regulate H3R function in general or after PAE is unknown, but one question is whether protein kinase-mediated capacity to desensitize H3Rs is diminished in PAE rats in a manner that may contribute to the heightened H3 receptor coupling we have observed in PAE rats.

Another mechanism for modulating H3R function that may be altered after PAE relates to receptor regulation by interactions with other transmitter receptors and other modulatory proteins. Consistent with our observations of rH_3A_ mRNA-overexpressing HEK-293 cells (Figure 9B), a number of studies have reported that H3Rs can dimerize with a variety of other neurotransmitter receptors (Shenton et al., 2005; Márquez-Gómez et al., 2018) as well as form trimers combining with dopamine D1 and NMDA-glutamate receptors (Rodríguez-Ruiz et al., 2017). Typically, these reports show how H3Rs modulate the function of its dimer / trimer partner, but do not report whether activation of the partner receptors alters H3R function. Whether or not PAE alters the heterodimerization of receptors or changes the function of H3R-containing dimers is unknown. However, one regulatory protein that may be of particular interest here is the Sigma 1 receptor (S1R) which acts as a “chaperone protein” modifying the activity of many protein targets, including GPCRs in membranes and cytoplasm of different cell types (Mishra et al., 2015; Pasternak, 2017), including H3Rs (Moreno et al., 2014). Curiously, many different classes of clinically-used neuroactive agents have representatives that, in addition to their primary mechanism of action, also act either as S1R agonists or antagonists (Cobos et al., 2008). One such class of agents is the H3R inverse agonists (Riddy et al., 2019; Szczepańska et al., 2021). Indeed, Riddy and colleagues reported that ABT-239, the agent that reversed both LTP and learning deficits in our PAE rats (Savage et al., 2010; Varaschin et al., 2010), has similar affinity for both H3Rs and S1Rs. As ABT-239 reversed PAE-induced LTP deficits without affecting LTP in control rats, one possible interpretation of this differential effect of ABT-239 may be a difference in how S1Rs modulate H3R function in PAE rats compared to control, a question currently under investigation in our laboratory.

## Data availability statement

The raw data supporting the conclusions of this article will be made available by the authors, without undue reservation.

## Ethics statement

The animal study was reviewed and approved by University of New Mexico Health Sciences Center Institutional Animal Care and Use Committee.

## Author contributions

SD, CV, and DS conceived and contributed to the experimental design of the study. KL, ER, SD, and DS generated moderate PAE rat offspring for the *in situ* hybridization studies. KL, SD, and DS conducted and analyzed the *in situ* hybridization studies in rat. SD harvested and transfected HEK-293 cells with different rH3 mRNA isoforms for western blotting studies, conducted the qRT-PCR studies in H3R transgenic mice, and processed them for the western blotting studies. SD and CV supervised the management of the H3R transgenic mouse colony. ER and DS conducted and analyzed the GTP binding studies in H3R transgenic mice. SD and DS harvested brain tissues and prepared the manuscript. All authors read and approved the submitted version of the manuscript.

## Funding

This work was supported by NIH-NIAAA 1 P50 AA022534.

## Conflict of interest

The authors declare that the research was conducted in the absence of any commercial or financial relationships that could be construed as a potential conflict of interest.

## Publisher’s note

All claims expressed in this article are solely those of the authors and do not necessarily represent those of their affiliated organizations, or those of the publisher, the editors and the reviewers. Any product that may be evaluated in this article, or claim that may be made by its manufacturer, is not guaranteed or endorsed by the publisher.

## Footnotes

^1^www.matrixscience.com

^2^www.waters.com
